# Supplementary material for: SARS-CoV-2 genomic characterization and clinical manifestation of the COVID-19 outbreak in Uruguay
Source: Emerg Microbes Infect. 2021 Jan 15;10(1):51–65. doi: 10.1080/22221751.2020.1863747 (PMC7832039; doi:10.1080/22221751.2020.1863747)
Supplement: Supplemental Material [file TEMI_A_1863747_SM5859.docx]

SARS-CoV-2 genomic characterization and clinical manifestation of the COVID-19 outbreak in Uruguay

**Supporting Information**

**Supplemental Methods**

***Sample collection, RNA extraction and diagnostic RT-PCR***

Naso-oropharyngeal swabs (NOS), broncho-alveolar lavages (BAL), naso-pharyngeal aspirates (NA) and tracheal aspirates (TA) were collected in viral transport media (VTM) (Centers for Disease Control and Prevention, CDC; VTM, SOP# DSR-052-03) from suspect cases with acute respiratory symptoms, travel history to affected countries, and/or contact with COVID-19 confirmed cases. Sampling was done between March 17^th^ and May 26^th^, 2020, and samples were referred to the laboratory for diagnosis.

RNA was extracted from 300 or 500 μl per sample (in VTM) both using the QIAsymphonyⓇ DSP Virus/Pathogen Mini or Midi kit (Qiagen), respectively, and confirmatory qualitative commercial RT-PCR kits were used for diagnosis and screening (depending on critical availability during the outbreak) (**Table 1** in the main text). The criteria to consider a case positive is specified in the commercial kits’ datasheets [1-3]. For the only kit without specified cut-off, i.e., the RealStar SARS-CoV-2 RT-PCR kit (Altona), our laboratory used a calculated cut-off threshold of 40. The TIB BIOMOL has a cycle cut-off threshold of 36 (for the E gene) and 39 (for the RdRp gene); the GeneFinder commercial kit has a unique cycle cut-off threshold of 40 for the three genes (N, E, and RdRp genes).

***Library preparation and sequencing***

For viral genome amplification, we used the Swift Normalase Amplicon SARS-CoV-2 Panel (SNAP, Swift Biosciences, whole viral genome single tube NGS assay, cat# SN-5XCOV296). First, isolated total RNA was converted to first-strand cDNA by random priming using the Superscript IV first-strand synthesis system (Invitrogen, cat# 180901050). Random priming was performed as follows: 65˚C for 5 min, cooled on ice followed by addition of mix to run cDNA first-strand synthesis 23˚C 10 min, 50˚C for 30 min, 80˚C for 10 min, and 4˚C hold. Ten microliters of first-strand cDNA were inputted into the two-step PCR reaction. The first step incorporates tiled primer pairs that target and enrich for the entire 29.9 kb COVID-19 viral genome (NCBI Reference Sequence NC_045512.2) during multiplex PCR. Multiplex PCR was run as follows: 98˚C 30 sec, four repeating cycles of 98˚C/ 10 sec, 60˚C/5 min, 65˚C/1 min. Eighteen repeating cycles of 98˚C/10 sec, 64˚C/1 min. The multiplexed samples were cleaned with 1x volume room temperature Ampure XP beads (Beckman Coulter, cat# A63882) for 5 min at room temperature, washed with 80% ethanol twice, and beads were resuspended in 17.4 µl of TE buffer (part of the swift kit). The indexing reagent mix was added to the resuspended beads and run in the indexing PCR reaction (each sample contains a unique i5 Illumina index) at 37˚C/20mins, 98˚C/30secs, eight repeating cycles of 98 ˚C/10 sec, 60˚C/30 sec, 66˚C/1 min.

After indexing PCR was completed, samples were cleaned with 1x volume Ampure XP beads (5 min bind time, 80% ethanol wash x2) and eluted in 30 µl of TE buffer. The final library products were run on Agilent TapeStation 2200 with high sensitivity DNA screen tape to verify the amplicon size of about 450 bp; each library was quantified by qPCR using the Kapa-Roche Library quant kit (Illumina, cat# KK4824) on the Bio-Rad cfx384 real-time system. Samples were normalized, pooled, and run on the Illumina NovaSeq 6000 system on a 300 cycle flow cell. Run metrics were paired-end 150 cycles with dual indexing reads. Both positive and negative control samples were also run during library prep but not sequenced. The negative control sample was water, and the positive control sample consisted of SARS-CoV-2 genomes (Twist Biosciences, cat# 102024) serially diluted to 100 viral copies mixed into 50 ng of Universal Human Reference RNA (Agilent, cat# 18091050).

***Sequence read processing***

Sequencing reads were demultiplexed with Illumina bcl2fastq v.2.20 requiring a perfect match to indexing barcode sequences. Illumina sequencing adapters were trimmed with Trimmomatic v.0.39 [4]. Reads were aligned using BWA v.0.7.17 [5] to a custom index containing human genome reference (GRCh38/hg38), including unscaffolded contigs and alternate references, plus the reference SARS-CoV-2 genome (NC_045512.2, wuhCor1). Reads were soft-clipped at the location of PCR primers using primerclip v.0.38 [6]. Variants were called using bcftools v.1.9:

| bcftools mpileup --redo-BAQ --adjust-MQ 50 --gap-frac 0.05 --max-depth 10000 --max-idepth 200000 --output-type u \|  bcftools call --ploidy 1 --keep-alts --multiallelic-caller -f GQ  Raw pileups were filtered using  bcftools norm --check-ref w --output-type u \|  bcftools filter -i "INFO/DP>=10 & QUAL>=10 & GQ>=99 & FORMAT/DP>=10" --SnpGap 3 --IndelGap 10 --set-GTs . --output-type u \|  bcftools view -i 'GT="alt"' --trim-alt-alleles |
| --- |

Viral sequences were generated by applying VCF files to the reference sequence using `bcftools consensus` with -m to mask sites below 4,000x coverage depth with Ns, and -m N to mask sites of ambiguous genotypes with N. Only sequences with >4,000x mean viral coverage depth, >23,000 bp unmasked sequence, and >0.3 of reads mapping to the viral genome were analyzed. The data processing pipeline source is available at [7].

***Phylogenetic analyses***

***IQ trees.*** Maximum likelihood trees were performed using the IQ-TREE XSEDE tool, multicore version 2.0.6, on the Cipres Science gateway [8]. The best substitution model was determined among 87 options using ModelFinder, as implemented in the Cipres gateway. According to the Bayesian Information Criterion (BIC), TIM+F+I was chosen as the best-fit substitution model. Support values were generated with 1,000 bootstrap replicates and the ultrafast bootstrapping method. Phylogenetic trees were visualized in Interactive Tree Of Life (iTOL) v.5 [9] or FigTree v.1.4.3 [10].

***Haplotype network analysis***

Genetic distance-based haplotype networks were created using DnaSP6 [11] and Population Analysis with Reticulate Trees (PopART) software [12-14]. Briefly, aligned fasta files with unique ten character codes and capped invariable ends were loaded, and ambiguous characters were replaced with N’s. Nexus and phylip haplotype files were generated considering sites containing gaps and missing data, and invariant sites were removed. An output file with all haplotype and polymorphism information was saved to support the generation of trait tables. The phylip haplotype file was imported as alignment into PopART together with location and/or mutation trait files, and median-joining networks created (epsilon = 0) with manually set trait colors.

***Mapping the distribution SARS-CoV-2 clades within Uruguay***

We investigated each of the SARS-CoV-2 clades’ spatial distribution resulting from independent introduction events in Uruguay, as detected using BEAST. In particular, we focused on the capital city, Montevideo, for which we had the densest sampling of genomic sequences. We investigated the geographic spread of identified clades and the detection of phylogenetic clusters in hospitals, nursing homes, and research institutes in Uruguay.

***Software Scripts and Visualization***

Correlograms were generated using the corrplot and RColorBrewer packages in program R [15] and Rstudio [16] as described recently [17]. Dendrograms were calculated using the dendPlot function and hclust method, or as implemented in the heatmap/complexheatmap packages in R. Annotations were done using heatmap, complexheatmap, and ggplot2 packages. Correlation network diagrams were generated in undirected mode in R and RStudio using ggraph, igraph, tidyverse, and ggplot2 packages, with clustering based on the igraph layouts graphopt or dh. Edges are weighted according to *P*-values (inversely). Edges are only shown if *P* < 0.05, and nodes without edges were removed. Nodes are sized according to the *r* values of the connecting edges. Edge bundling graphs were generated in undirected mode in R and RStudio using ggraph, igraph, tidyverse, and RColorBrewer packages. Edges are only shown if *P* < 0.05, and nodes are sized according to the connecting edges’ r values. For lollipop plots, sequence retrieval and data manipulations were performed using dplyr (tidyverse) and seqinr packages, followed by data visualization using ggplot2.

Volcano plots were generated in Prism. Mirror bar charts were created in Microsoft Excel 2016. Multi-categorical alluvial diagrams were generated using RawGraphs with automatic sorting and 0.5 link opacity [18]. Stream graphs were created in RawGraphs using Expand representation as offset and Basis spline interpolation. Geocoding was done with the ggmap library in R/RStudio. Geographical contour heat maps with point annotations were generated using the packages ggmap, ggplot2, and dplyr.

**Supplemental References**

1. Altona. RealStar® SARS-CoV-2 RT-PCR Kit RUO 2020. Available from: <https://altona-diagnostics.com/en/products/reagents-140/reagents/realstar-real-time-pcr-reagents/realstar-sars-cov-2-rt-pcr-kit-ruo.html>.

2. GeneFinder. GeneFinder™ COVID-19 Plus RealAmp Kit 2020. Available from: <https://www.fda.gov/media/137116/download>.

3. Roche. Molecular diagnostic solutions from Roche - SARS-CoV-2 tests 2020. Available from: <https://www.roche.de/res/content/11630/sars-cov-2_tests_ubersichtsbroschure_en__1_.pdf>.

4. Bolger AM, Lohse M, Usadel B. Trimmomatic: a flexible trimmer for Illumina sequence data. Bioinformatics. 2014 Aug 1;30(15):2114-20.

5. Li H, Durbin R. Fast and accurate short read alignment with Burrows-Wheeler transform. Bioinformatics. 2009 Jul 15;25(14):1754-60.

6. Swift Biosciences. Primerclip 2020. Available from: <https://github.com/swiftbiosciences/primerclip>.

7. NYU-sequencing-core. Codes used in viral sequence data processing 2020. Available from: <https://github.com/mauranolab/mapping/tree/master/dnase>.

8. Miller MA, Pfeiffer W, Schwartz T, editors. Creating the CIPRES Science Gateway for Inference of Large Phylogenetic Trees. Gateway Computing Environments Workshop; 2010; New Orleans, LA.

9. Letunic I, Bork P. Interactive Tree Of Life (iTOL) v4: recent updates and new developments. Nucleic Acids Res. 2019 Jul 2;47(W1):W256-W259.

10. Rambaut A. FigTree. 1.4. Edinburgh, UK: 2012.

11. Rozas J, Ferrer-Mata A, Sanchez-DelBarrio JC, et al. DnaSP 6: DNA Sequence Polymorphism Analysis of Large Datasets. Mol Biol Evol. 2017;34(12):3299-3302.

12. Bandelt HJ, Forster P, Rohl A. Median-joining networks for inferring intraspecific phylogenies. Mol Biol Evol. 1999 Jan;16(1):37-48.

13. Leigh J, Bryant D. PopART: Full-feature software for haplotype network construction. Methods Ecol Evol. 2015;6(9):1110–1116.

14. PopART. Population Analysis with Reticulate Trees (PopART) 2020. Available from: <http://popart.otago.ac.nz>.

15. Team RC. R: A language and environment for statistical computing and graphics. Vienna, Austria: 2013.

16. RStudio. RStudio Team: a bundle of RStudio’s popular professional software for statistical data analysis, package management, and sharing data products. Boston, MA: RStudio, Inc.; 2015.

17. Tuen M, Bimela JS, Banin AN, et al. Immune Correlates of Disease Progression in Linked HIV-1 Infection. Front Immunol. 2019;10:1062.

18. Mauri M, Elli T, Caviglia G, et al., editors. RAWGraphs: A Visualisation Platform to Create Open Outputs. 12th Biannual Conference on Italian SIGCHI Chapter; 2017; Cagliari, Italy: Association for Computing Machinery, New York, NY.

**Figures**


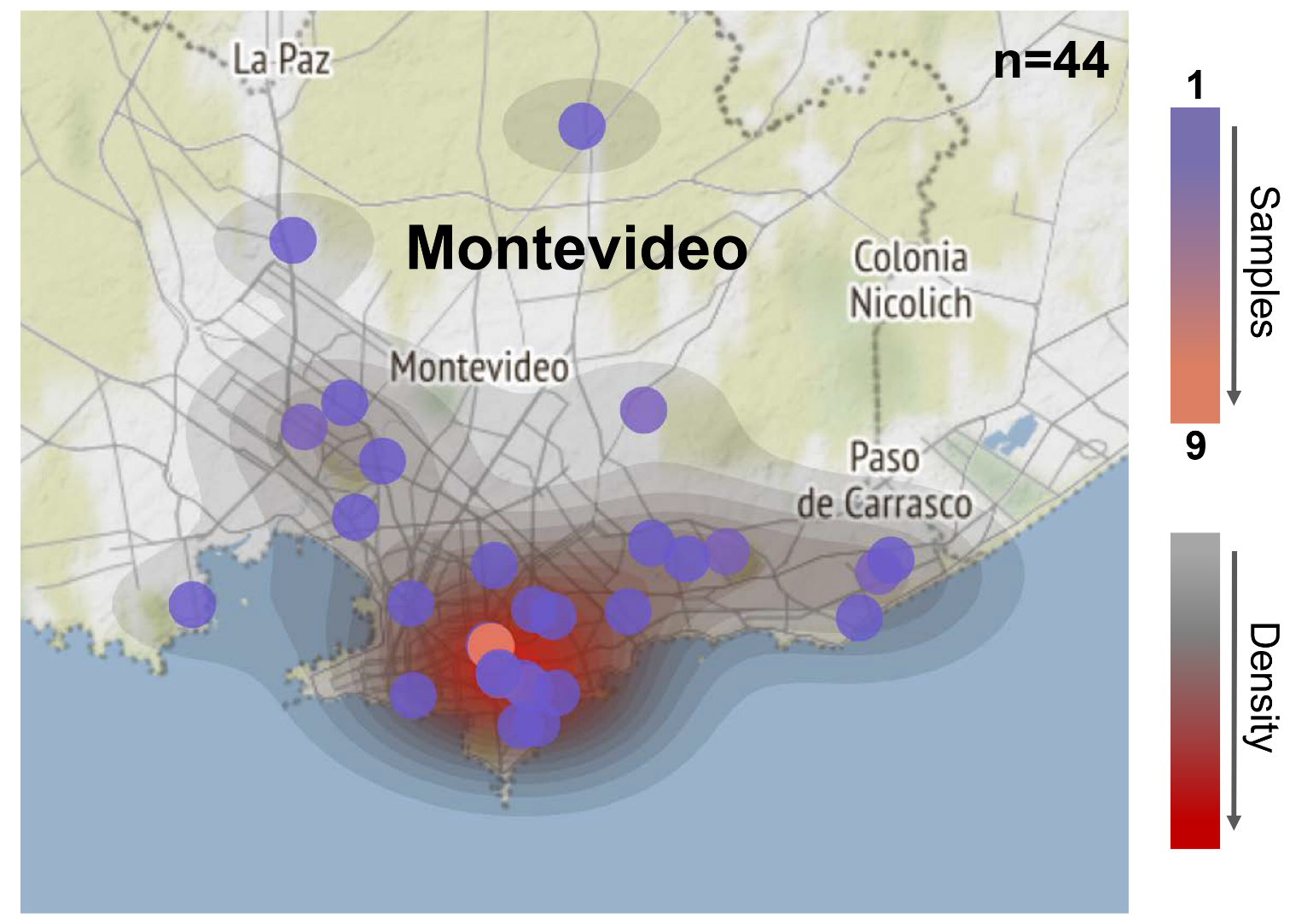


**Figure S1. Map of SARS-CoV-2 sample collection in our Uruguayan study cohort.**

Map of Montevideo, Uruguay, with sampling locations indicated by dots that are colored by sample numbers according to the legend to the right. Sampling density was most significant around the center of Montevideo, indicated by a gray-to-red density gradient.

**
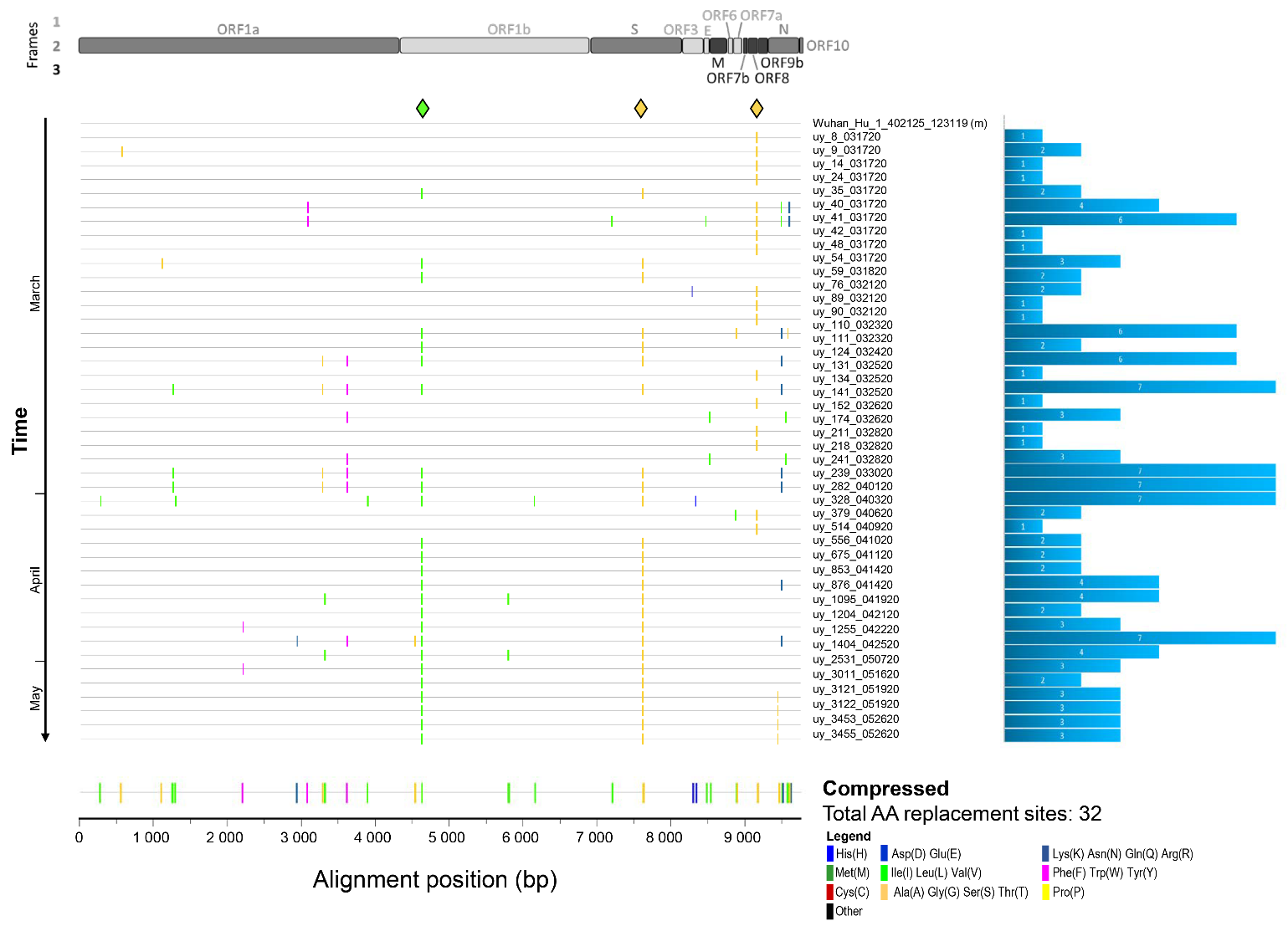
**

**Figure S2. SARS-CoV-2 amino acid replacements over time.**

Highlighter plot showing amino acid (AA) replacements of Uruguayan study sequences compared to the reference Wuhan-Hu-1 sequence as master (on top). Mutations are shown as ticks, color-coded according to the legend at the bottom. Study sequences are sorted along the y-axis according to sampling time, with the earliest sequences on top and most recent sequences at the bottom. A SARS-CoV-2 genome map is shown on top with protein regions consecutively assembled as done for the AA alignment. The gray tones of the protein bars relate to the reading frames of their coding genes. All occurring AA replacements are summarized at the bottom of the plot (compressed). AA replacements that are prevalent in >30% of study sequences are indicated by diamonds on top of the plot in the color of the replacing amino acid. A bar chart is shown to the right summarizing the number of AA replacements per study sample, aligned with the highlighter results and sample IDs on the left.

**
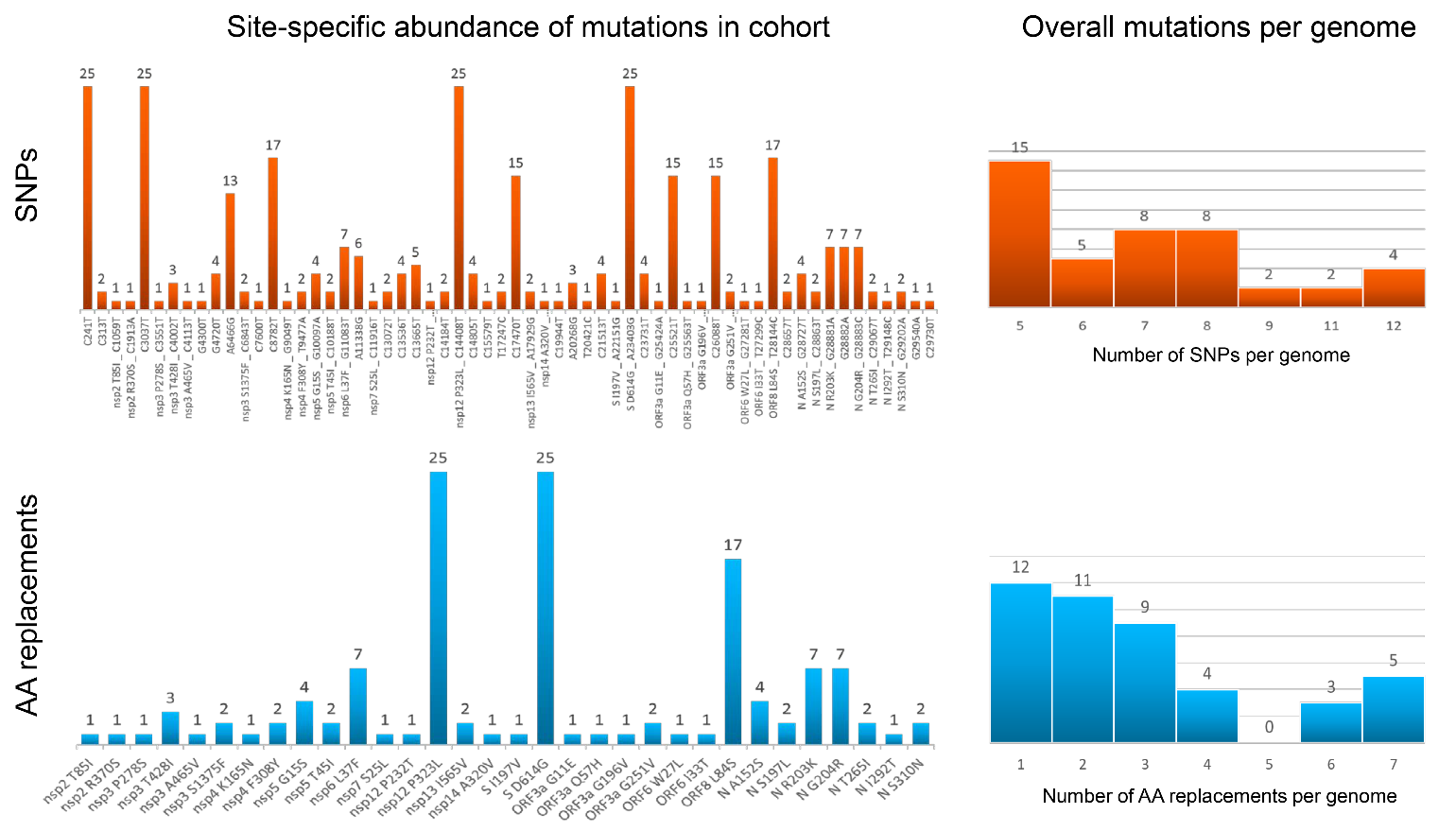
**

**Figure S3. Summary of SARS-CoV-2 BP and AA mutations in the cohort and per subject.**

**Left**: Bar diagrams showing mutation counts per site in the entire cohort. All single nucleotide polymorphisms (SNPs) are shown on the upper left, with total counts indicated on the y-axis and as numerical values on top of each bar. The base pair (BP) mutations are indicated on the x-axis together with the amino acid (AA) replacement, if applicable. On the lower left, all AA replacements are shown separately. **Right**: Bar diagrams showing cohort-wide mutation counts per genome. SNPs are shown on the upper right, AA replacements on the lower right. The number of mutations per genome is listed on the x-axis, and the occurrences in the cohort on the y-axis and as numerical values on top of each bar.

**
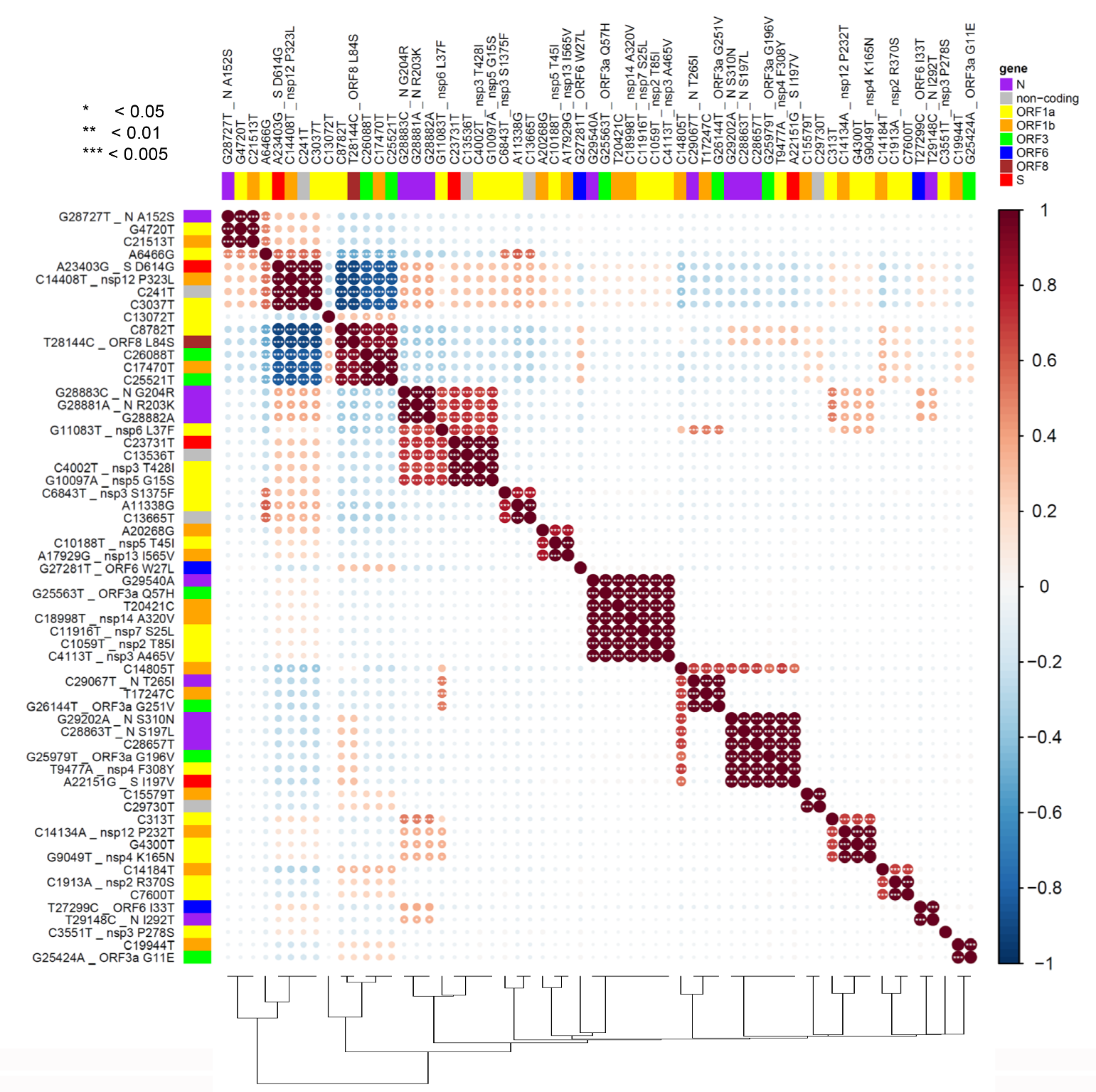
**

**Figure S4. Uruguayan SARS-CoV-2 mutation clusters.**

Correlogram summarizing associations of mutations observed in our Uruguayan study cohort (n=44) with squares sized and color-coded according to the magnitude of the correlation coefficient (*r*). The color code of *r* values is shown to the right; red colors represent positive, blue colors negative correlations between two connected parameters on the x- and y-axes. Asterisks indicate statistically significant correlations (*P < 0.05, **P < 0.01, ***P < 0.005). The correlogram is shown with hierarchical clustering according to the dendrogram at the bottom. The color-strip indicates gene relatedness of mutations according to the color code in the legend. Correlation analysis was done using non-parametric Spearman rank tests.

**
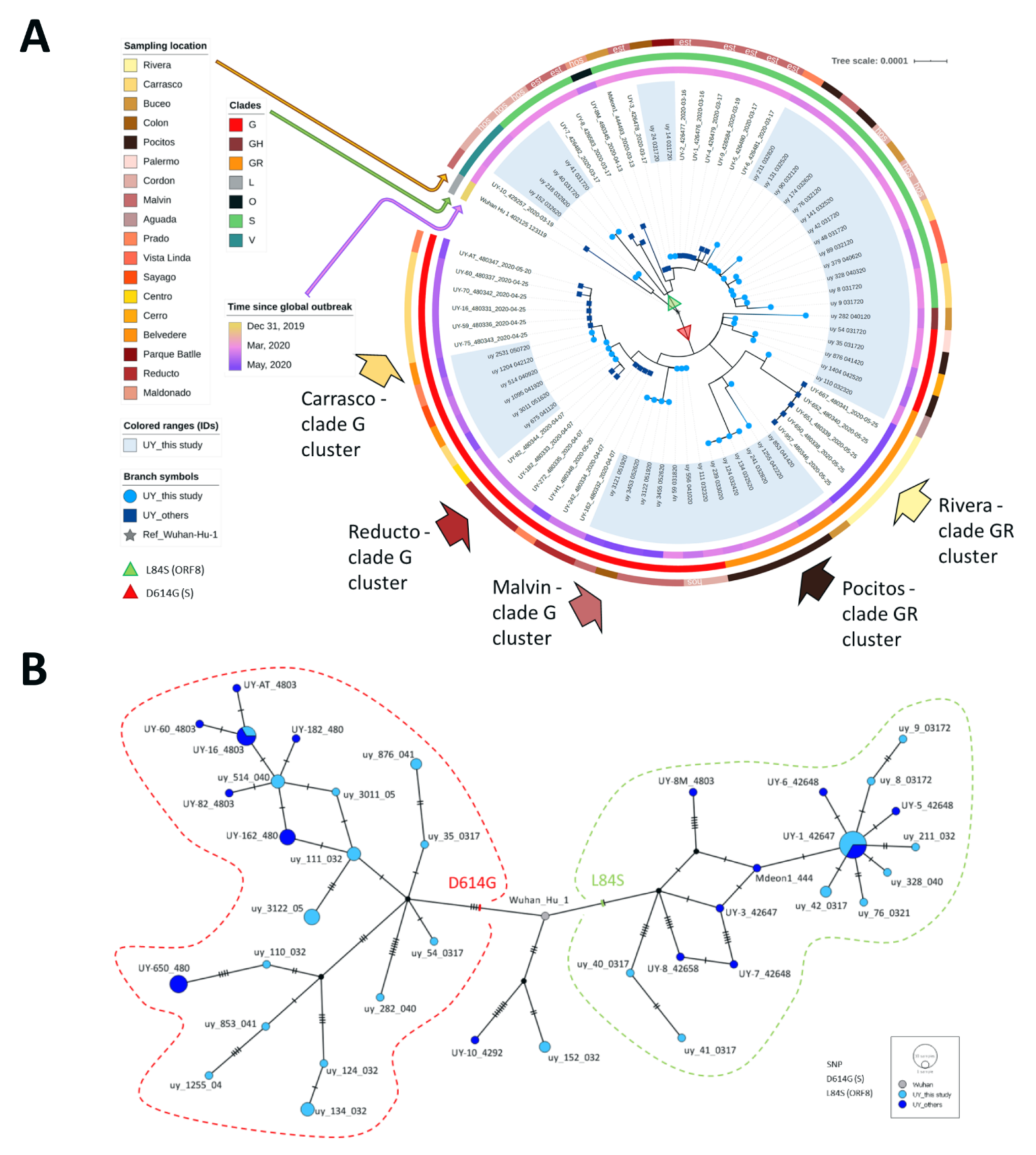
**

**Figure S5. Phylogenetic and mutation network analysis of Uruguayan SARS-CoV-2 viruses.**

**A.** Maximum-likelihood IQ-tree of 73 Uruguayan SARS-CoV-2 sequences and Wuhan-Hu-1 reference sequence, run with 1,000 Bootstrap replications. Branch symbols and taxa (ID colored ranges) are colored according to country and study source, as explained in the figure legend. The introductions of key mutations are shown by red and green triangles. For each sample, reported/estimated site of infection/sampling location (est: estimated, hos: treating hospital), clades, and time since global outbreak are indicated by the circular color strip around the tree according to the legend. Clustered appearances of clades at specific sites/neighborhoods are highlighted by arrows and labeled. **B.** Genetic distance-based haplotype network analysis of Uruguayan SARS-CoV-2 sequences. Circles represent populations of sequences with identical mutation patterns (haplotypes) as compared to Wuhan-Hu-1 as reference (gaps and missing data not considered). The circles are sized and colored relative to the number and source countries of contributing sequences, respectively. One sample ID annotates each haplotype population representatively. Ticks on the connecting lines indicate discriminating mutations between haplotypes. Two major branch-defining mutations are highlighted as red and green ticks according to the legend. Red and green polygons encircle haplotype populations carrying respective key mutations (spike D614G and ORF8 L84S).


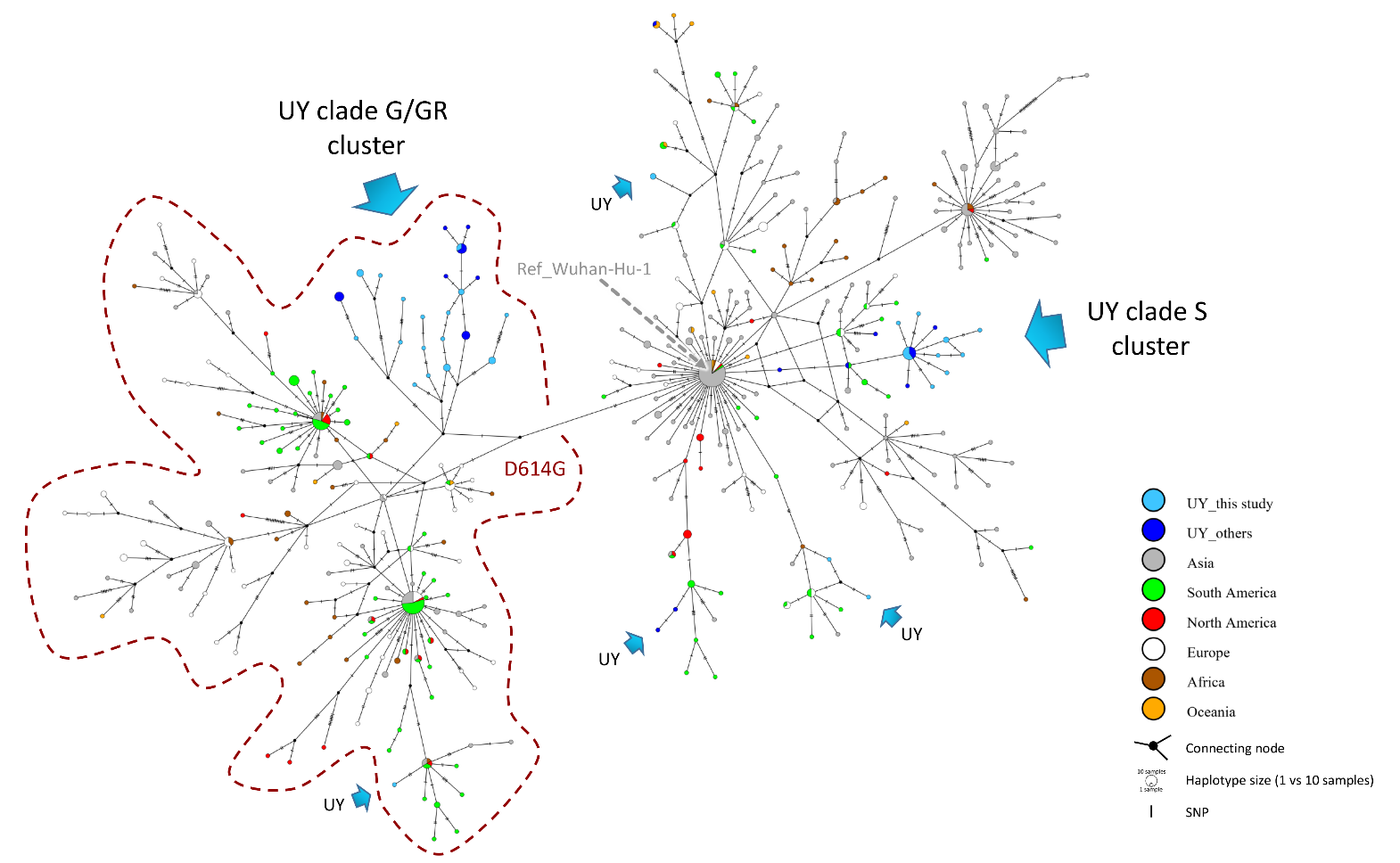


**Figure S6. Haplotype network analyses of Uruguayan SARS-CoV-2 sequences among global reference strains.**

Genetic distance-based haplotype network analysis of 73 Uruguayan SARS-CoV-2 sequences among 609 subsampled, global SARS-CoV-2 sequences. Circles represent populations of sequences with identical mutation patterns (haplotypes) as compared to Wuhan-Hu-1 as reference (gaps and missing data not considered). The circles are sized and colored relative to the number and source countries of contributing sequences, respectively. One sample ID annotates each haplotype population representatively. Ticks on the connecting lines indicate discriminating mutations between haplotypes. The node carrying the Wuhan-Hu-1 reference sequence is labeled, and haplotypes sharing the spike D614G mutation among the sequences of the reticular network are encircled by a dashed, dark red polygon. The location of major and minor clusters of Uruguayan sequences is highlighted by large and small blue arrows, respectively.

**
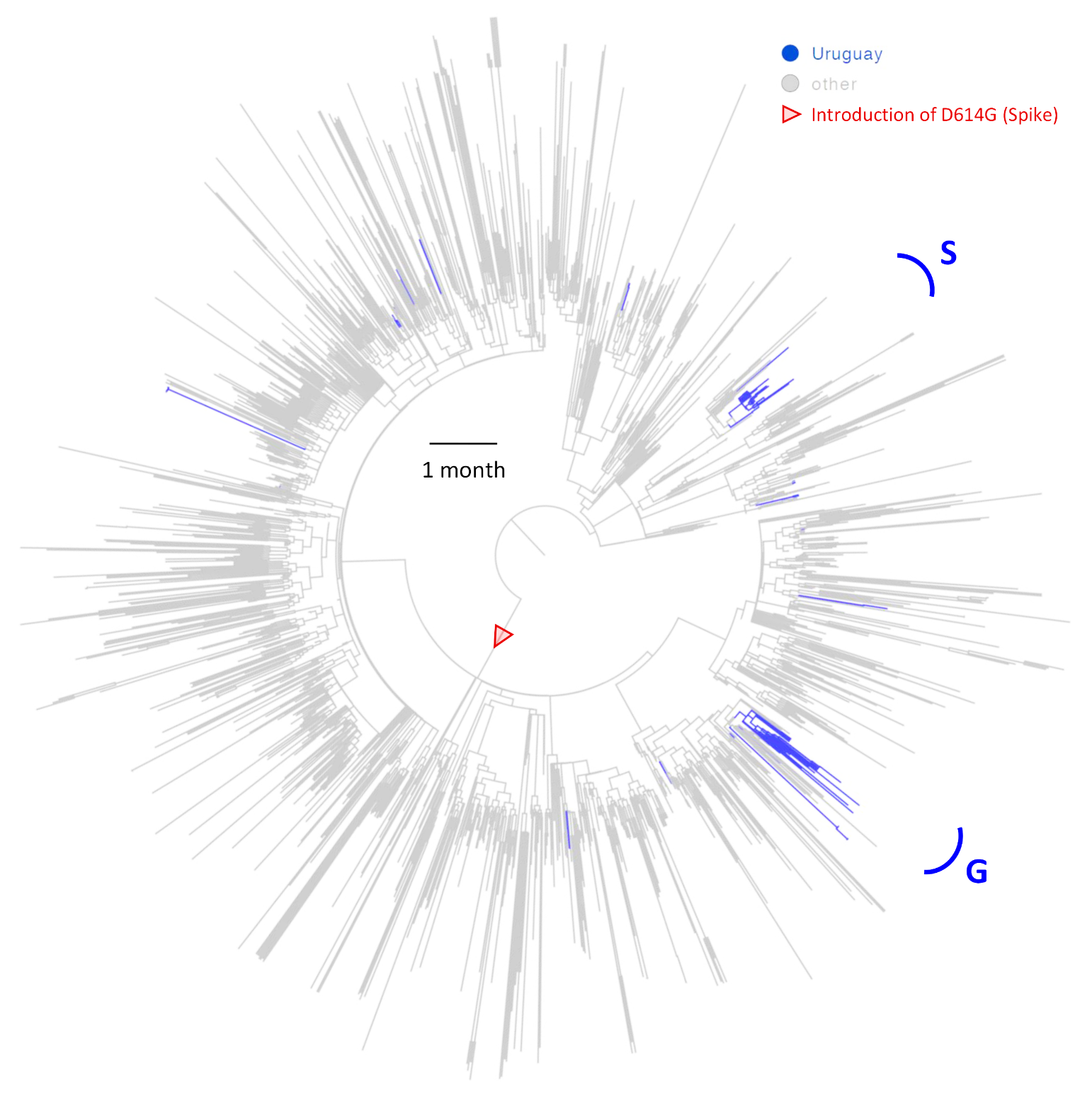
**

**Figure S7. Time-scaled phylogenetic tree to identify Uruguayan clusters.**

A cluster is defined as a phylogenetic clade corresponding to an independent introduction into Uruguay. Uruguayan sequence tree branches are colored blue and non-Uruguayan sequence branches gray. The two main clusters are highlighted by brackets, and their GISAID clades are indicated. The introduction of the spike D614G mutation is indicated by a red arrowhead.

**
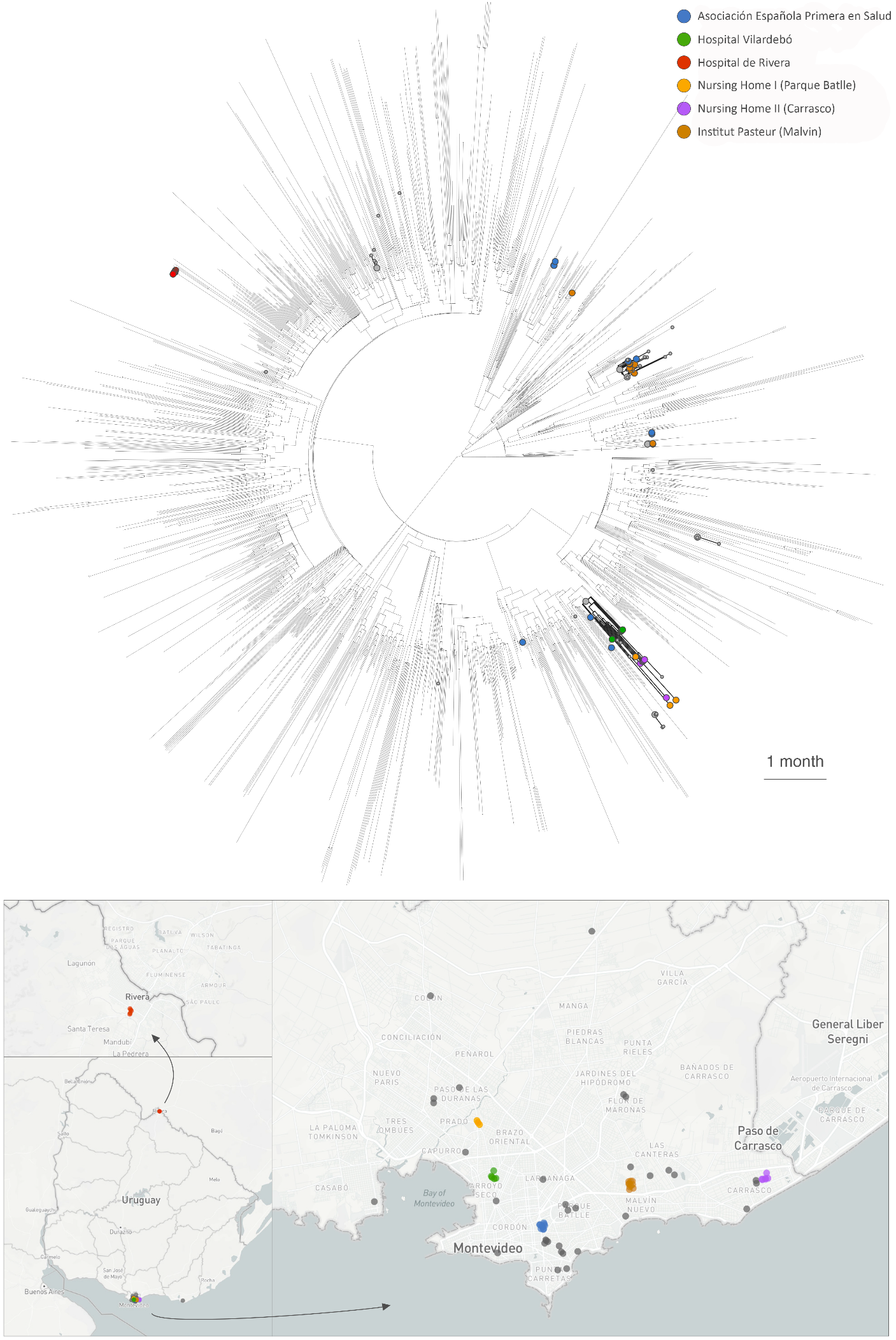
**

**Figure S8. A visualization of the evolutionary relationships and spatial distribution of our SARS-CoV-2 samples in hospitals and nursing homes in Montevideo.**

Time-scaled maximum clade credibility tree (MCC) generated by the discrete phylogeographic analysis of 1,810 SARS-CoV-2 sequences. According to the legend, Uruguayan sequences are shown as colored circles both in the phylogenetic tree and in the Uruguayan maps, color-coded based on their relationship to hospitals, nursing homes, and research institutes. Samples that are not associated with a hospital, nursing home, or research institute are shown as gray circles.

**
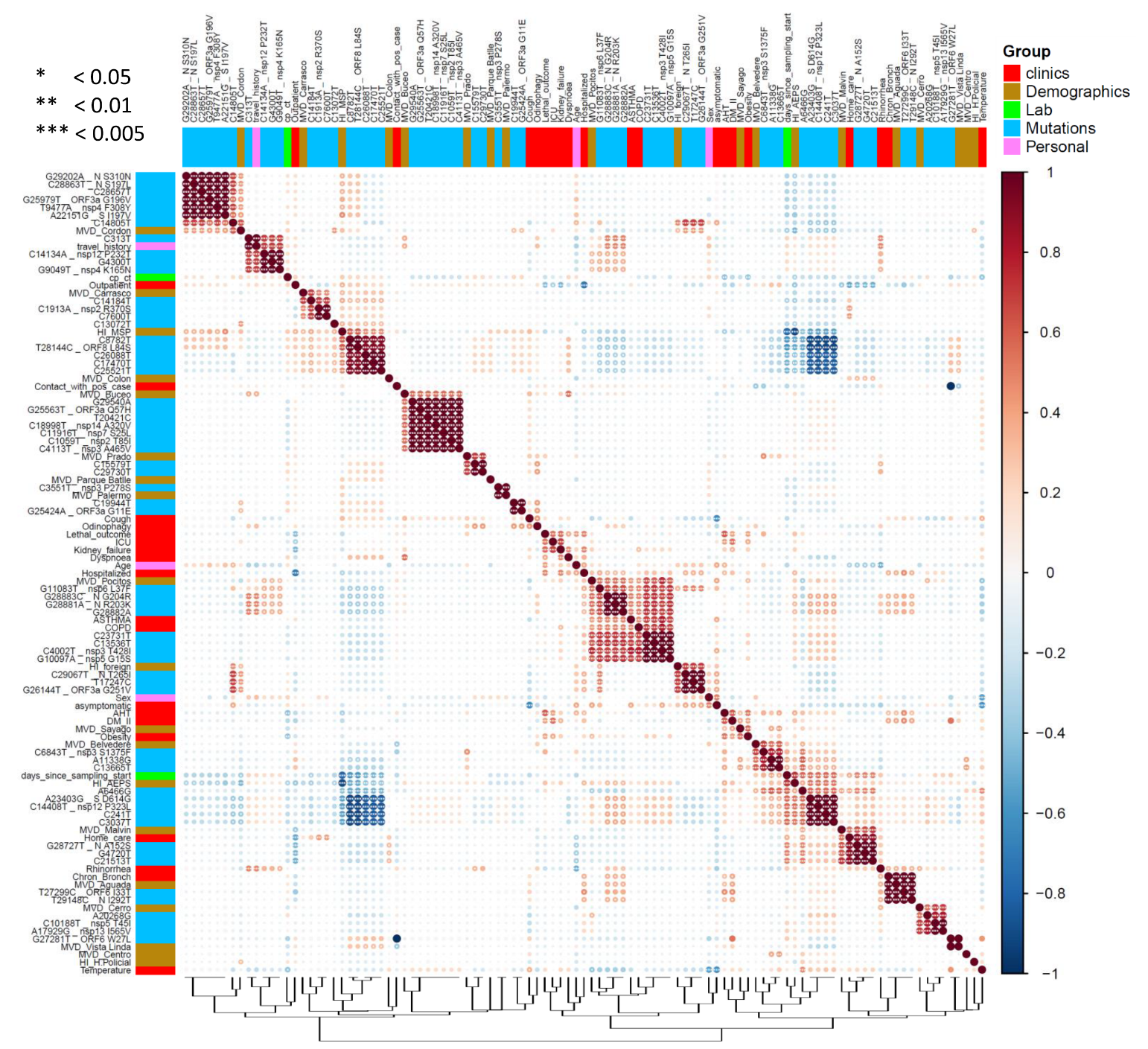
**

**Figure S9. Correlation analysis of viral, demographic, and clinical parameters of Uruguayan study samples/participants.**

Correlogram summarizing associations among indicated parameters in our Uruguayan study cohort (n=44) with squares sized and color-coded according to the magnitude of the correlation coefficient (*r*). The color code of *r* values is shown to the right; red colors represent positive, blue colors negative correlations between two connected parameters on the x- and y-axes. Asterisks indicate statistically significant correlations (*P < 0.05, **P < 0.01, ***P < 0.005). The correlogram is shown with hierarchical clustering according to the dendrogram at the bottom. The color-strip indicates group relatedness of parameters according to the color code in the legend. Correlation analysis was done using nonparametric Spearman rank tests. MVD: Montevideo, HI: healthcare institution.

**
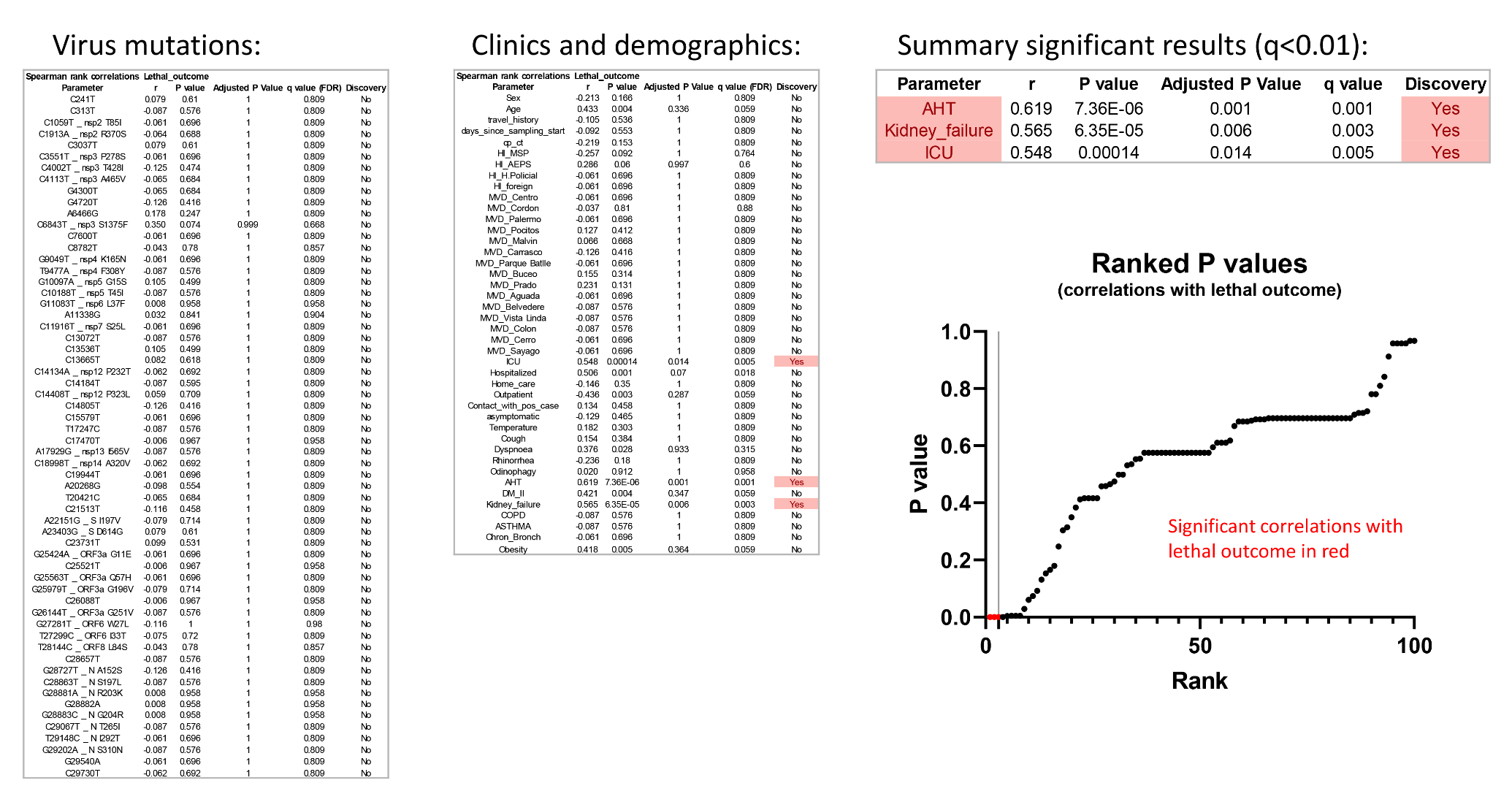
**

**Figure S10. Statistics of correlations with lethal outcome of SARS-CoV-2 infection in our Uruguayan study cohort.**

Correlation statistics between lethal outcome and virus mutations are shown on the **left**, and between lethal outcome and clinical and demographic parameters in the **middle**. A summary of significant correlations, according to *q*<0.01, is shown on the **upper right**. For each correlation, correlation values *r*, *P* values, adjusted *P* values, and false discovery rates (FDR) *q* values are displayed together with significant discovery assessment. The distribution of *P* values across the data set is illustrated in a dot plot of ranked *P* values in the **lower right**. All significant results are highlighted in red. MVD: Montevideo, HI: healthcare institution, ICU: intensive care unit, AHT: arterial hypertension, DM II: diabetes mellitus type II, COPD: chronic obstructive pulmonary disease.

**
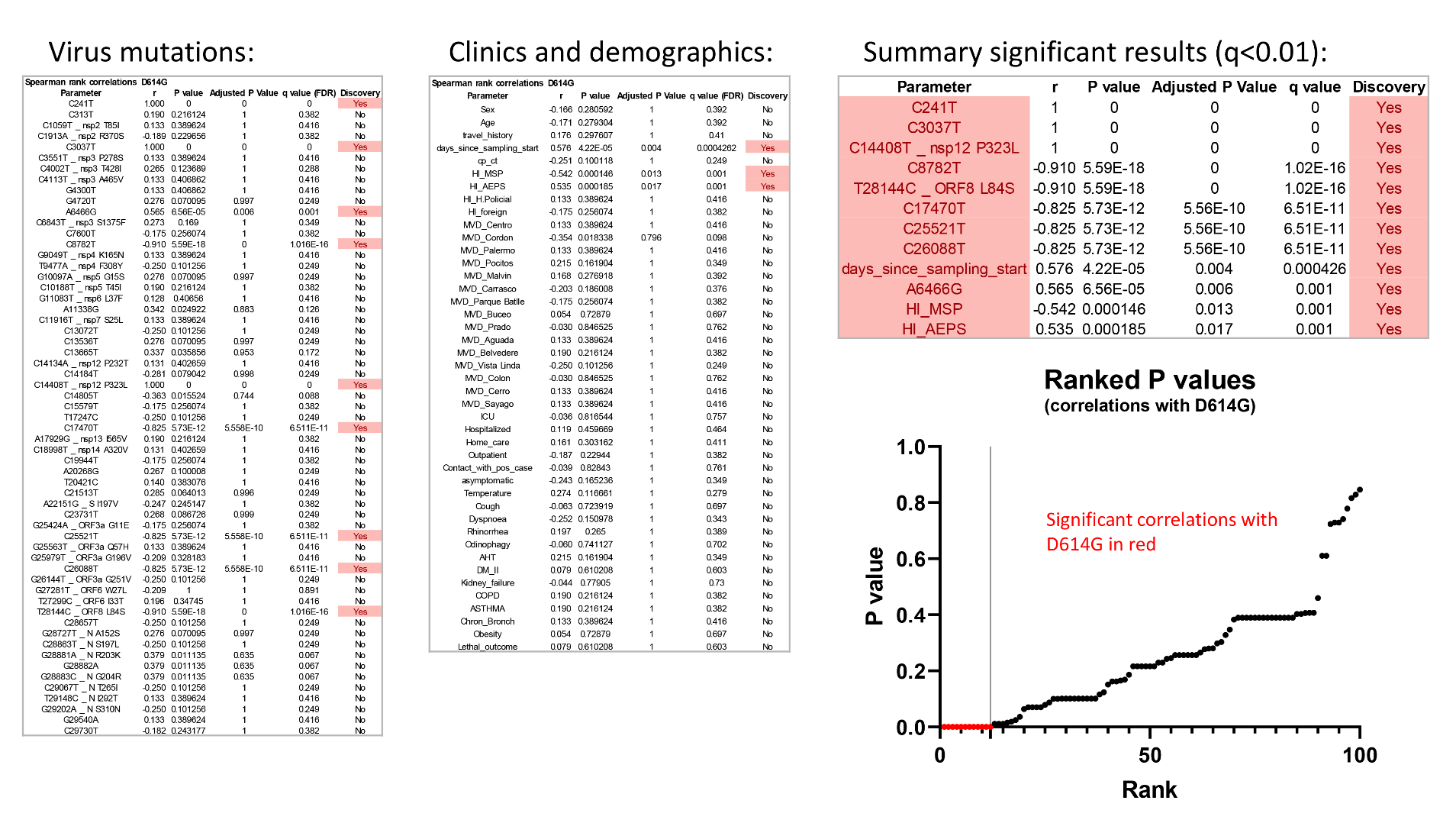
**

**Figure S11. Statistics of correlations with D614G spike mutation in infecting viruses of our Uruguayan study cohort.**

Correlation statistics between the presence of D614G spike mutation and other SARS-CoV-2 mutations are shown on the **left**, and between D614G spike mutation and clinical and demographic parameters in the **middle**. A summary of significant correlations, according to *q*<0.01, is shown on the **upper right**. For each correlation, correlation values *r*, *P* values, adjusted *P* values, and false discovery rates (FDR) *q* values are displayed together with assessment of significant discovery. The distribution of *P* values across the data set is illustrated in a dot plot of ranked *P* values in the **lower right**. All significant results are highlighted in red. MVD: Montevideo, HI: healthcare institution, ICU: intensive care unit, AHT: arterial hypertension, DM II: diabetes mellitus type II, COPD: chronic obstructive pulmonary disease.
